# Circulating Pulmonary-Originated Epithelial Biomarkers for Acute Respiratory Distress Syndrome: A Systematic Review and Meta-Analysis

**DOI:** 10.3390/ijms24076090

**Published:** 2023-03-23

**Authors:** Huishu Lin, Qisijing Liu, Lei Zhao, Ziquan Liu, Huanhuan Cui, Penghui Li, Haojun Fan, Liqiong Guo

**Affiliations:** 1 Institute of Disaster and Emergency Medicine, Tianjin University, Tianjin 300072, China; linhuishu99@outlook.com (H.L.); zhaolei0130@163.com (L.Z.); liuziquan@tju.edu.cn (Z.L.); 2 Wenzhou Safety (Emergency) Institute, Tianjin University, Wenzhou 325000, China; 18322610302@163.com; 3 Tianjin Key Laboratory of Disaster Medicine Technology, Tianjin 300072, China; 4 Research Institute of Public Health, School of Medicine, Nankai University, Tianjin 300381, China; liuqisijing@nankai.edu.cn; 5 School of Environmental Science and Safety Engineering, Tianjin University of Technology, Tianjin 300384, China; lipenghui406@163.com

**Keywords:** acute respiratory distress syndrome, acute lung injury, biomarker, pulmonary-originated proteins

## Abstract

Previous studies have found several biomarkers for acute respiratory distress syndrome (ARDS), but the accuracy of most biomarkers is still in doubt due to the occurrence of other comorbidities. In this systematic review and meta-analysis, we aimed to explore ideal ARDS biomarkers which can reflect pathophysiology features precisely and better identify at-risk patients and predict mortality. Web of Science, PubMed, Embase, OVID, and the Cochrane Library were systematically searched for studies assessing the reliability of pulmonary-originated epithelial proteins in ARDS. A total of 32 studies appeared eligible for meta-analysis, including 2654 ARDS/ALI patients in this study. In the at-risk patients’ identification group, the highest pooled effect size was observed in Krebs von den Lungren-6 (KL-6) (SMD: 1.17 [95% CI: 0.55, 1.79]), followed by club cell proteins 16 (CC16) (SMD: 0.74 [95% CI: 0.01, 1.46]), and surfactant proteins-D (SP-D) (SMD: 0.71 [95% CI: 0.57, 0.84]). For the mortality prediction group, CC16 exhibited the largest effect size with SMD of 0.92 (95% CI: 0.42, 1.43). Meanwhile, the summary receiver operating characteristic (SROC) of CC16 for ARDS diagnosis reached an AUC of 0.80 (95% CI: 0.76, 0.83). In conclusion, this study provides a ranking system for pulmonary-originated epithelial biomarkers according to their association with distinguishing at-risk patients and predicting mortality. In addition, the study provides evidence for the advantage of biomarkers over traditional diagnostic criteria. The performance of biomarkers may help to clinically improve the ARDS diagnosis and mortality prediction.

## 1. Introduction

Acute respiratory distress syndrome (ARDS) has been reported to be a major problem in intensive care units (ICU), with mortality of 40% worldwide [1,2,3,4]. Currently, the diagnosis of ARDS and acute lung injury (ALI) is based on the American European Consensus Conference (AECC) criteria and the Berlin definition, which centers around the cut-off of the PaO_2_/FiO_2_ ratio [5,6]. However, the current diagnosis is criticized as having high sensitivity but low specificity [7].

The Berlin definition partially overcomes some restrictions of the AECC definition [8]. Still, two-thirds of ARDS patients experienced delayed or missed diagnosis with the recognition rate ranging from 51% in mild patients to 79% in severe patients [1]. The accuracy of ARDS is now being questioned, and experts have called for additional criteria to supplement the clinical definition [7].

Accumulating evidence suggests that baseline biomarkers can help distinguish ARDS patients and predict mortality [9,10,11,12,13] since the biomarkers can reflect alterations in pathophysiological characters at the molecular level and provide relevant diagnostic evidence. A previous meta-analysis proposed a ranking system for ARDS plasma biomarkers [14]. Most of them focus on the inflammatory factors, while critically ill patients are universally confronted with other comorbidities, such as sepsis, which will result in a significant increase in inflammatory factors which compromise the specificity of inflammation biomarkers [15].

Diffuse alveolar damage is the central pathological process of ARDS, characterized by alveolar–capillary membrane damage and hyperpermeable alveolar edema, which induce the alveolar type II cell injury and dysfunction in the synthesis and metabolism of surfactant proteins, thus leading to small airway trapping and alveolar atrophy atelectasis [16]. Severely damaged bronchial cells also cannot be ignored during lung injury [17]. Numerous clinical studies have focused on circulating proteins reflecting alveolar epithelial injury, surfactant proteins (SP-A/B/C/D) [18,19,20], Krebs von den Lungren-6 (KL-6) [21,22,23], and club cell proteins (CC10/16) specifically [24,25,26]. In the process of ARDS development, the capacity of the surfactant to lower surface tension is impaired since surfactant function is inhibited by protein in the oedema fluid [27]. In addition, elevated serum KL-6 levels have been previously reported in ARDS patients which may due to the increased leakage of KL-6 from the alveolar space into the circulation [28]. Meanwhile, the increased permeability of the alveolocapillary membrane in ARDS patients leads to increased plasma levels of the club cell protein [26]. The soluble receptor for advanced glycation end-products (sRAGE) is also an important biomarker reflecting lung epithelial injury. A previous, individual patient data (IPD) meta-analysis illustrated its correlation with 90-day mortality [29]. IPD meta-analysis is believed to be the gold standard analytical approach in meta-analysis [30]. Therefore, even though RAGE is closely related to our topic, it has not been discussed in this study.

Biomarkers might be an important part of ARDS’ diagnosis, which could assist in identifying ARDS at-risk patients, and predicting mortality. This study aims to provide a quantitative overview of biomarkers by comparing the concentration of biomarkers between ARDS/at-risk patients and survivors/non-survivors. In addition, the diagnostic value between traditional criteria and biomarker is compared. To conclude the effect size of the current study, we conducted a systematic review and meta-analysis of continuous variables to assess biomarkers in the same way as previous approaches [14,31]. The Berlin/AECC definition was set as the comparison criteria in the diagnostic test accuracy meta-analysis and applied to investigate the advantage of biomarkers. 

## 2. Materials and Methods

### 2.1. Study Design

This systematic review and meta-analysis was conducted under the guidance of Preferred Reporting Items for Systematic Reviews and Meta-Analyses (PRISMA) Statement [32].

### 2.2. Search Strategy

Web of Science, PubMed, Embase, OVID, and Cochrane Library were systematically searched from inception to 31 December 2021 to identify all studies reporting target proteins. A total of 8612 articles in related fields were yielded. Search strategies applied are presented in detail in Additional file 3.

### 2.3. Study Selection

The included criteria of this study were formulated by all authors in accordance with the principle of PICOS (population, intervention, control, outcomes). The PICOS of the current study were as follow: patients diagnosed as ARDS/ALI by a medical professional, regardless of definition. The control group should be at-risk patients or matched controls who shared similar characteristics. Healthy controls were rigorously excluded because an ARDS’ delayed or missed diagnosis was predominantly identified among critically ill patients. The endpoints were defined as the ARDS at-risk patients who developed ARDS for the diagnostic prediction group and the death of ARDS patients for the mortality prediction group. Primary screening was performed by two independent researchers (H.L. and Q.L.) by titles and abstracts. The exclusion criteria were as follows: (1) non-research articles: reviews, conference abstracts, books, case reports, methodological articles, etc.; (2) animal and/or in vitro studies; (3) not relevant field (some abbreviations are also widely used outside the medical field, leading to the retrieval of irrelevant documents); (4) no target protein; (5) infant population; (6) treatment outcome. Potential articles were further confirmed by a full-text reading. The exclusion criteria were an article that (1) did not include outcome of interests; (2) did not include population of interest; (3) conference abstract; (4) insufficient data; (5) review and commentary; (6) case report; (7) full-text access not available; (8) written in a language other than Chinese or English. A third researcher (L.G.) was consulted when a disagreement arose. 

### 2.4. Data Extraction

Two researchers (H.L. and Q.L.) completed the data extraction independently. The baseline biomarker level was defined as the first record in the study in co-authors’ agreement unless particular statements were made. The mean and standard deviation or standard error were extracted for meta-analysis of continuous variables. Data presented as median and quantiles were transformed into mean and standard deviation by a mathematical approach [33,34]. Accuracy of biomarker diagnostic tests were performed when more than five studies fulfilled the following criteria: (1) studies based on the AECC or Berlin definition; (2) true positive (TP), true negative (TN), false positive (FP), false negative (FN), sensitivity (Se), specificity (Sp), accuracy, positive predictive value (PPV), or negative predictive value (NPV) were reported. Data were also extracted when those variables could be calculated from known variables (Se and Sp). Moreover, demographic variables (study design, setting, study population, ARDS (ALI) definition, study size, the number of ARDS/ALI patients, age, gender, plasma sample moment) were recorded.

### 2.5. Risk of Bias

The quality of included studies was evaluated with the revised Quality Assessment of Diagnostic Accuracy Studies tool (QUADAS-2) as previous biomarker meta-analyses have reported [31,35]. Part of the contents was adjusted to better fit the study. The signaling question in patient selection, “Was a case-control design avoided?” was replaced by “Were the specific inclusion and exclusion criteria mentioned?”, because it was inappropriate to neglect case-control studies in our study design. In index tests, the question of “If a threshold was used, was it pre-specified?” would not be answered unless studies were included for diagnostic test accuracy. Risk of bias was conducted via Review Manager version 5.3 (Cochrane Collaboration, Oxford, UK) and relevant figures are presented in Figure S1 (Supplementary Materials). More details were presented in Additional file 2.

### 2.6. Statistical Analysis

#### 2.6.1. Meta-Analysis of Continuous Variables

Standardized mean difference (SMD) using Cohen’s d was employed to assess the effect size for continuous variables, and 95% confidence intervals (CIs) were calculated. SMD is the quotient of the difference between the two means and the pooled standard deviation, which eliminates the effect of different units in the analysis [36]. SMD is a relative indicator with good consistency, which can better combine research results for further analysis. Articles provided with the number of ARDS patients and non-ARDS patients were categorized as the diagnosis group, while those providing the survivors and non-survivors were called the prognosis group to predict mortality. Subgroup analysis of SP-D was based on studies with similar etiological characters, namely pneumonia and sepsis to assess potential heterogeneity. Another subgroup analysis was performed in a group with more than two studies with the Berlin/AECC definition to find heterogeneity between different diagnostic criteria. Heterogeneity among included studies was evaluated by I^2^. A random effect model was applied when I^2^ > 50%. I^2^ > 50% is considered as medium heterogeneity and >75% is considered as high heterogeneity [37]. Biomarkers with more than three studies are presented as forest plots. Sensitivity analyses were performed by removing each study and recalculating the pooled effect size to explore the consistency of the included study and seek potential bias for biomarkers with more than two studies. Funnel plots and Egger regression were employed to check for publication bias. The significance level in this meta-analysis was settled as *p* < 0.05 and <0.1 in Egger regression, respectively. All statistical analyses were performed with Stata 17 (Stata Corp LLC, College Station, TX, USA).

#### 2.6.2. Meta-Analysis of Diagnostic Test Accuracy

The diagnostic score and diagnostic odds ratio (DOR) were present in the forest plot, and 95% CI was calculated. DOR is the ratio of the odds of the TP relative to the odds of FP, which can provide a pooled measurement of a diagnostic test. We plotted a summary receiver operating characteristic (SROC) and calculated area under the curve (AUC) to examine the diagnostic accuracy. The likelihood ratio is a comprehensive index which reflects the sensitivity and specificity and provides a well-rounded diagnostic value. A posterior probability plot was created based on likelihood via GraphPad Prism 9.4 (GraphPad Software, La Jolla, CA, USA). Sensitive analysis and Deek’s funnel were applied to determine potential bias. All statistical analyses were performed with Stata 17 (Stata Corp LLC, College Station, TX, USA).

## 3. Results

### 3.1. Literature Search

**Figure 1:**
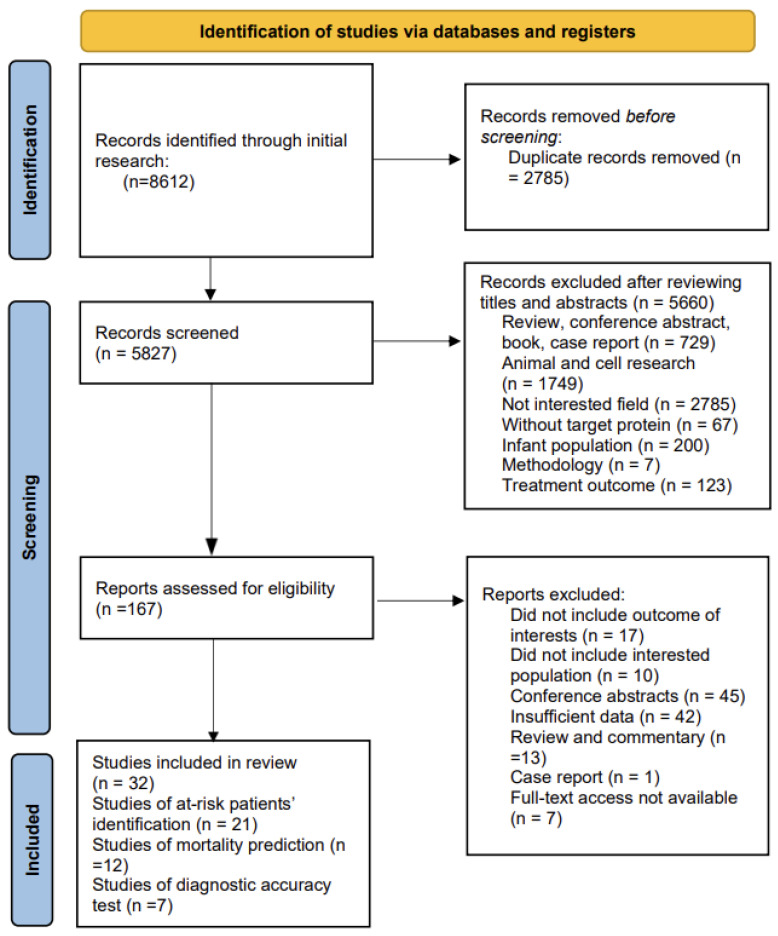
PRISMA flow diagram of literature searching and inclusion.

In the initial search, 8612 articles were identified (PubMed: 1119; Web of Science: 2013; OVID: 2492; Embase: 2869; Cochrane Library: 119). After the removal of duplicates, the remaining 5827 articles were screened for the title and abstract. Based on the inclusion and exclusion criteria, 167 studies were assessed for full-text eligibility. A total of 32 studies were eligible for data extraction and included in this meta-analysis (Figure 1). 

### 3.2. Study Characteristics and Quality Assessment

Demographic characters of diagnosis prediction, mortality prediction, and diagnostic test accuracy are presented in Table 1, Table 2 and Table 3. In total, 2654 ARDS/ALI patients were involved in this study. In a meta-analysis of continuous variables, 22 studies were included for diagnosis prediction (SP-A: 4 studies [18,19,38,39]; SP-B: 2 studies [19,38]; SP-D: 11 studies [9,11,20,26,40,41,42,43,44,45,46]; KL-6: 5 studies [9,22,23,26,41]; CC16: 8 studies [10,13,26,41,44,47,48,49,50]) and 12 studies for mortality prediction (SP-D: 7 studies [9,11,12,44,50,51,52]; KL-6: 3 studies [9,12,21]; CC16: 5 studies [10,13,52,53,54]). In a meta-analysis of diagnostic test accuracy, seven studies of CC16 were included [13,25,26,44,48,49,55]. All the included studies were based on ELISA to detect the concentration of biomarkers, although the detection units were inconsistent. ARDS/ALI diagnosis was based on the AECC/Berlin definition in 25 studies and 4 studies were based on the derived AECC/Berlin definition (AECC/Berlin plus other clinical criteria). Other diagnosis standards were lung injury score and comprehensive clinical index because some studies had been carried out relatively earlier. The sampling time varied from the time of diagnosis to within 48 h of ICU admission. Most patients were critically ill and some presented with complications. Raw data of ARDS patients extracted from original article for further analysis are listed in Tables S1–S3.

The Berlin/AECC was viewed as the gold test in ARDS diagnosis. Only studies based on the AECC/Berlin definition or derived AECC/Berlin definition were included for the CC16 diagnostic accuracy test. The AUC of included studies ranged from 0.60 to 0.91. 

Detailed ARDS etiology is presented in Table S4 in the Supplementary Materials. Because some studies did not provide specific etiological characteristics, the total number of patients in this table is less than the total number of patients included. Therefore, there will be non-negligible heterogeneity. Pneumonia is the most common cause of ARDS, with sepsis ranking second. ARDS patients also more frequently encounter trauma and aspiration. The subgroup analysis of SP-D was based on studies with similar etiological characters, namely pneumonia and sepsis. Some studies simultaneously referred to more than one biomarker or reported diagnosis, prognosis, and AUC. These studies are analyzed in each circumstance.

The most predominant concern that arose from the QUADAS-2 quality assessment was the patient selection. Some of the studies did not provide detailed inclusion and exclusion standards. The overall quality of the included studies was satisfactory. 

### 3.3. Biomarkers Associated with ARDS at-Risk Patients’ Identification

**Figure 2:**
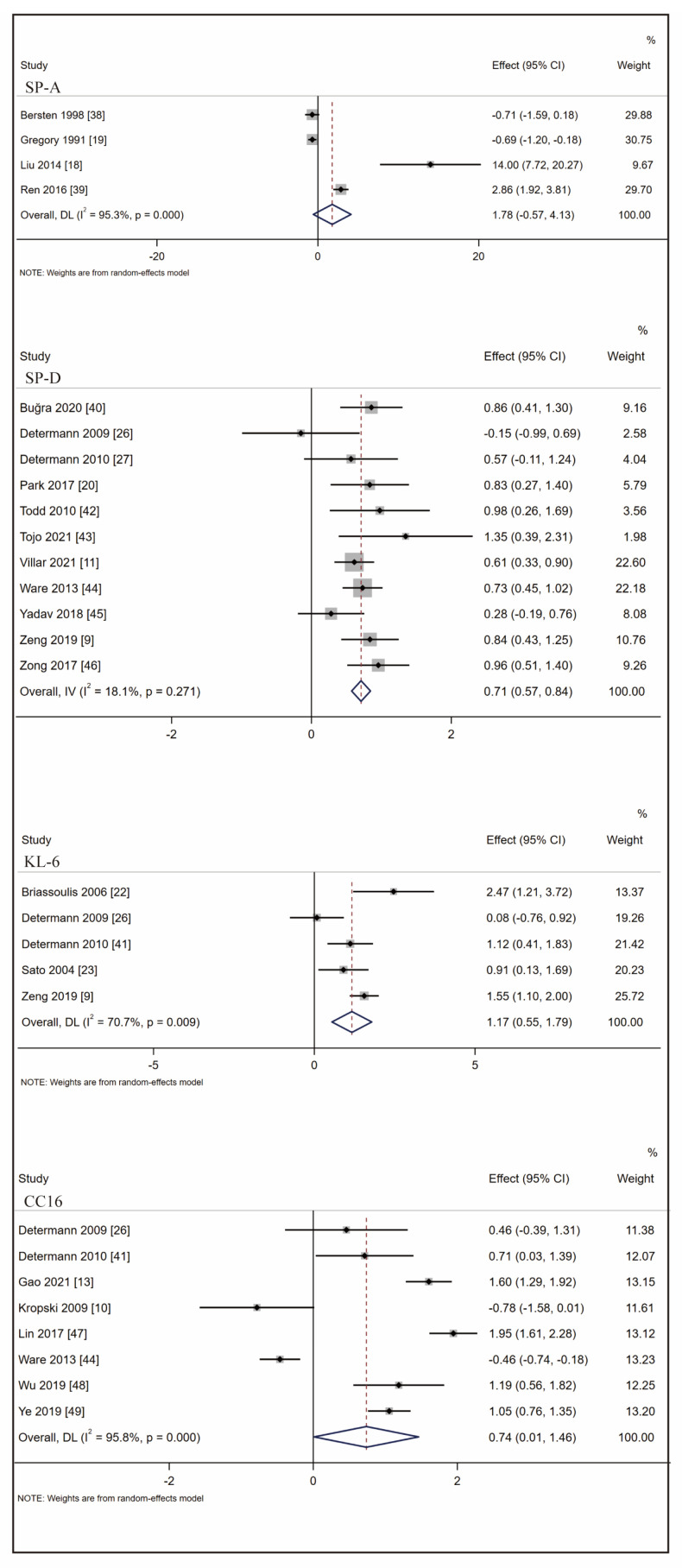
Forest plot of SMD for the association between SP-A, SP-D, KL-6, CC16, and ARDS identification. (Note: SMD: standardized mean difference; SP-A: surfactant protein A; SP-D: surfactant protein D; KL-6: Krebs von den Lungren-6; CC16: club cell protein 16; ARDS: acute respiratory distress syndrome).

We performed meta-analysis on biomarkers with identified clinical studies (SP-A/B/D, KL-6, and CC16) to explore the association in identifying ARDS among at-risk patients. Forest plots were created for biomarkers with more than three studies (SP-A/D, KL-6, CC16). Only two studies were found for SP-B, and the effect size was not significant (SMD: 0.28 [95% CI: −0.72, 1.28]). The combined effect of SP-A is not significant either because a large proportion of the research was close to the invalid line, and exhibited different trends. The overall effect size ranged from 0.72 to 1.17 among biomarkers with significant SMD. The highest pooled effect size was observed in KL-6 with SMD of 1.17 (95% CI: 0.55,1.79), followed by CC16 (SMD: 0.74 [95% CI 0.01, 1.46]) and SP-D (SMD: 0.71 [95% CI: 0.57, 0.84]) (Figure 2). Although the effect size exhibited the identification capacity of biomarkers, the heterogeneity cannot be disregarded. To conclude, SP-D showed the lowest heterogeneity and KL-6 was the most valuable biomarker in at-risk patients’ identification.

**Figure 3:**
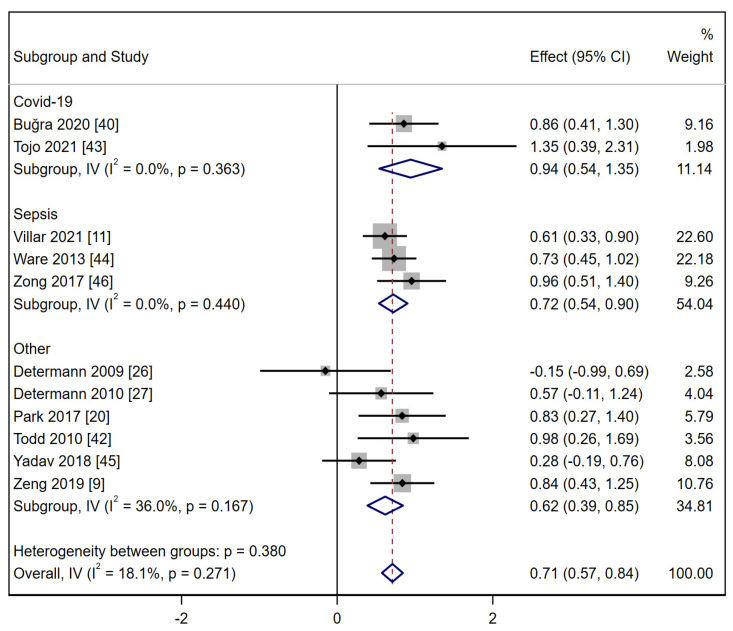
Forest plot of SMD for the association between SP-D subgroup and ARDS identification. (Note: SMD: standardized mean difference; SP-D: surfactant protein D; ARDS: acute respiratory distress syndrome).

Subgroup analysis was performed in the SP-D group, in which some included studies were based on particular comorbidity, including COVID-19, sepsis, and other comorbidities (Figure 3). The pooled effect size was 0.94 (95% CI: 0.54, 1.35) for COVID-19 with the ARDS group and 0.72 (95% CI: 0.54, 0.90) for sepsis with ARDS. Even though the heterogeneity was low for the SP-D group, I^2^ decreased significantly in these two subgroups.

Sensitivity analyses were performed to explore the consistency of included studies and seek potential bias (Table S5). By removing each study and recalculating the pooled effect size, the heterogeneity might be dominated by the limited number of studies. A subgroup study was performed in SP-D/KL-6/CC16 to find heterogeneity between diagnostic criteria. I^2^ decreased in CC16 when the subgroup was classified as AECC or Berlin definition (Figure S2). 

### 3.4. Biomarkers Associated with ARDS Mortality Prediction

**Figure 4:**
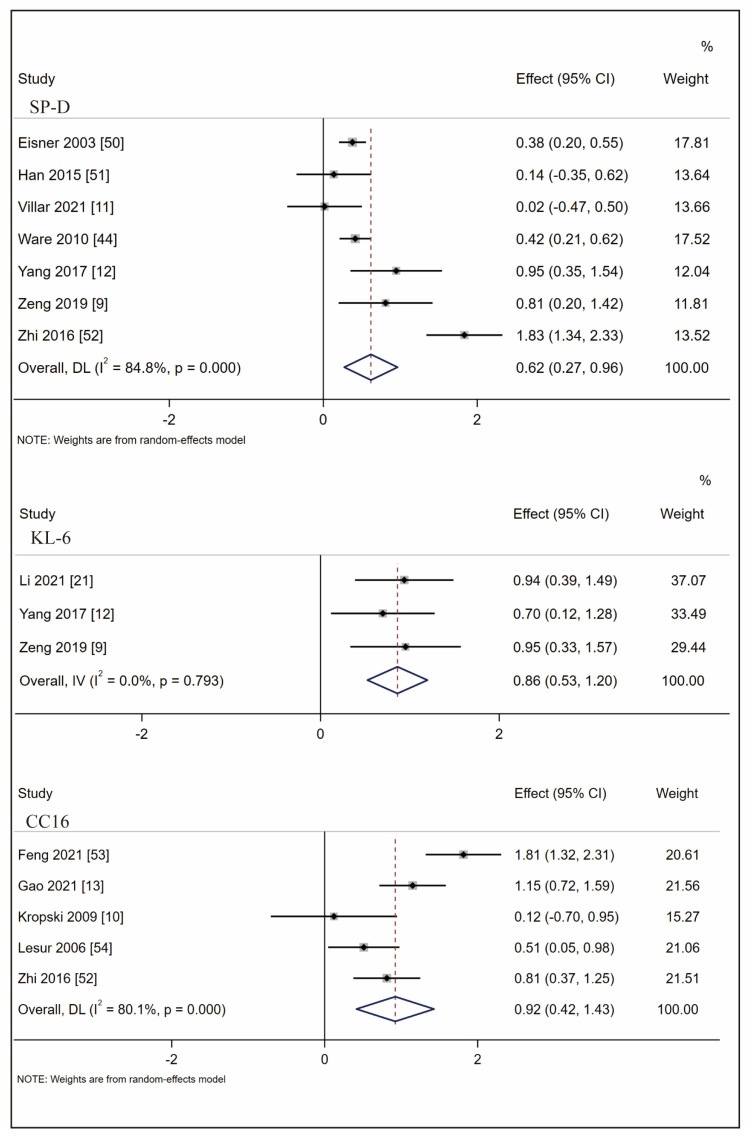
Forest plot of SMD for the association between SP-D, KL-6, CC16, and ARDS mortality. (Note: SMD: standardized mean difference; SP-D: surfactant protein D; KL-6: Krebs von den Lungren-6; CC16: club cell protein 16; ARDS: acute respiratory distress syndrome).

We performed meta-analyses on biomarkers with identified clinical studies (SP-A/D, KL-6, and CC16) to explore the association with ARDS mortality. Forest plots were created in biomarkers with more than three studies (SP-D, KL-6, CC16). Only two studies were found for SP-A, and the effect size was not significant (SMD: −0.42 [95% CI: −1.39, 0.55]). As shown in Figure 4, the effect size was 0.62 (95% CI: 0.27, 0.96) for SP-D, 0.86 (95% CI: 0.53, 1.20) for KL-6, and 0.92 (95% CI: 0.42, 1.43) for CC16. To conclude, KL-6 showed the lowest heterogeneity and CC16 is the most valuable biomarker in mortality prediction.

Sensitivity analyses were performed to explore the consistency of included studies and seek potential bias (Table S6). In the SP-D group, a study (Zhi 2016) brought visible heterogeneity which increased the pooled effect size from 0.40 to 0.62. A subgroup study was performed in SP-D/CC16 to find heterogeneity between diagnostic criteria. I^2^ decreased in SP-D/CC16 when subgroup was classified as the Berlin definition (Figure S3).

### 3.5. CC16 Diagnosis Test Accuracy

**Figure 5:**
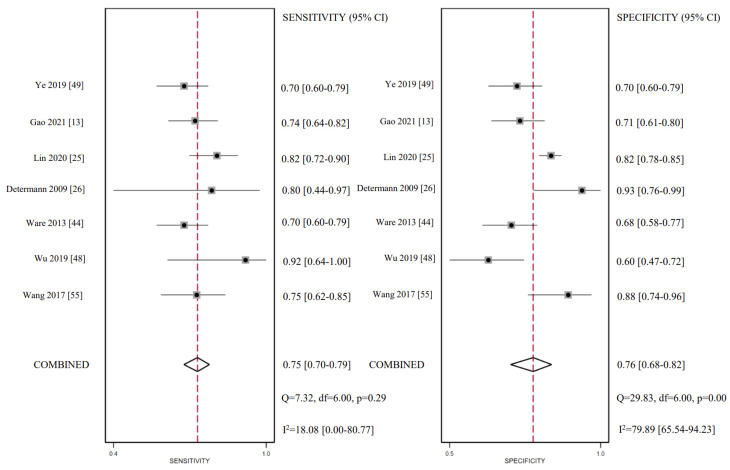
Forest plots of sensitivity and specificity for CC16.

**Figure 6:**
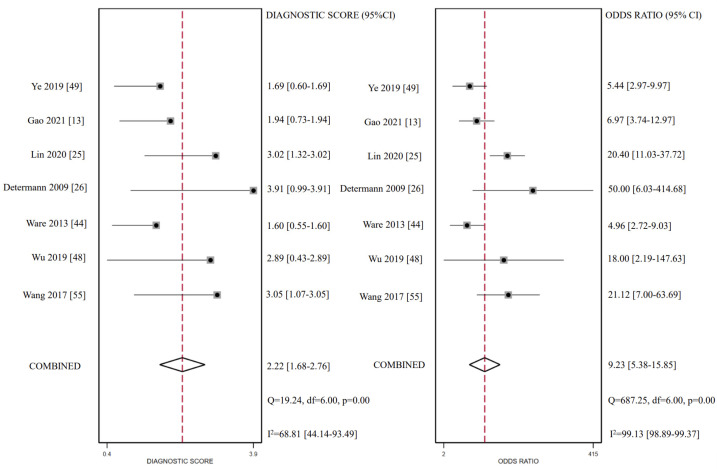
Forest plot for diagnostic score and odds ratio for CC16.

**Figure 7:**
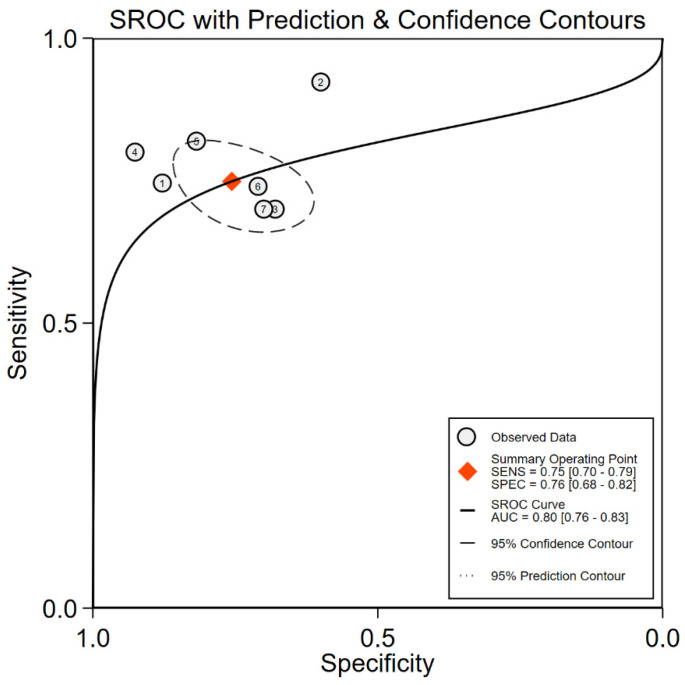
Summary receiver operating characteristic curves (SROC) for CC16.

**Figure 8:**
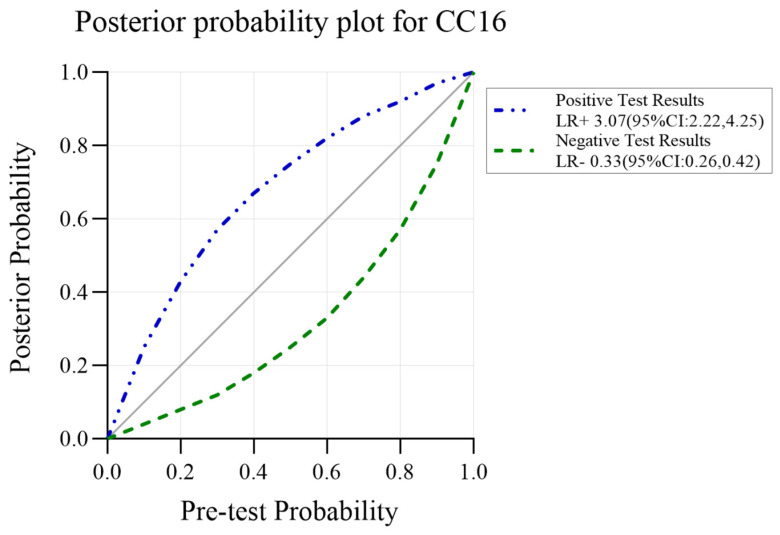
Posterior probability plot for CC16.

A diagnostic test accuracy meta-analysis can better explain the clinical value of biomarkers. Unfortunately, it was unable to take diagnostic tests in other biomarkers due to the limitation in the quantities of studies. Seven studies provided data on the CC16 diagnostic value on ARDS diagnosis. The pooled sensitivity was 0.75 (95% CI: 0.70, 0.79) and specificity was 0.76 (95% CI: 0.68, 0.82). However, substantial statistical heterogeneity was present for specificity (I^2^ = 79.89). The diagnostic score was 2.22 (95% CI: 1.68, 2.76) and the diagnostic odds ratio was 9.23 (95% CI: 5.38, 15.85), while the heterogeneity remained high. All estimates corresponded to LR + 3.07 (95% CI: 2.22, 4.25) and LR-0.33 (95% CI: 0.26, 0.42). CC16 for ARDS diagnosis reached an AUC of 0.80 (95% CI: 0.76, 0.82). Fagan’s nomogram was created to evaluate the clinical utility of CC16 and concluded in a posterior probability plot (Figure 5, Figure 6, Figure 7 and Figure 8). Sensitivity analysis indicated that no study exhibited significant heterogeneity (Figure S4). 

### 3.6. Publication Bias

Funnel plots were performed for biomarkers in continuous variables meta-analysis with more than three studies (Figures S5 and S6) and the Egger’s test was applied to detect significant publication bias. From observation, funnel plot analysis indicated minor asymmetry. However, the Egger’s test demonstrated that there was no statistically significant publication bias (Table S7). In the CC16 diagnostic accuracy test, no bias was detected to be publication bias after being assessed by Deek’s’ funnel plot (Figure S7). 

## 4. Discussion

We conducted the updated meta-analysis of circulating pulmonary-originated epithelial biomarkers of ARDS and the first diagnostic test accuracy for CC16 in ARDS. Our study demonstrated that SP-D, KL-6, and CC16 are promising biomarkers for identifying ARDS at-risk patients and predicting mortality. 

In this study, Cohen’s d was proposed as the effect size (SMD) and employed to rank the biomarkers of continuous variables. Cohen’s d of 0.2, 0.5, and 0.8 are considered as small, medium, and large differences between groups [56]. In at-risk patients’ identification, the Cohen’s d were 0.71, 0.74, and 1.17 for SP-D, CC16, and KL-6, respectively. In mortality prediction, the Cohen’s d were 0.62, 0.86, and 0.92 for SP-D, KL-6, and CC16, respectively. Although significant increases were observed in our study, results should be interpreted cautiously due to the limited number of included studies. The diagnostic value of CC16 was also confirmed in the study, which exhibited a pooled sensitivity of 75% and a specificity of 76% and significantly increased the diagnostic accuracy. After considering the heterogeneity, it was inappropriate to rank these three biomarkers. Some studies explored the combined diagnostic value of ARDS biomarkers, which exhibited higher sensitivity and specificity than a single biomarker [13,44]. Therefore, future studies are suggested to further explore the combined diagnostic value of these biomarkers.

Acute lung irritants’ exposure has been reported to cause elevated CC16 in the serum [57]. Previous pioneering work by Terpstra et al. reported that CC16 was not significant to be a biomarker either for ARDS diagnosis or for mortality prediction [14]. With the accumulation of studies, these results would not remain unchanged, and meta-analysis should be updated at an appropriate interval. In this study, CC16 continuous variable meta-analysis reported a Cohen’s d of 0.74 (95% CI: 0.01, 1.46) in at-risk patients’ identification and ranked first in mortality prediction. In the diagnostic accuracy test, CC16 exhibited the possibility of shifting the pre-test probability of 50% to the post-test probability of 75%. This study is believed to provide strong evidence for considering CC16 as a potential biomarker for ARDS. 

Previous meta-analyses on ARDS biomarkers generally included inflammatory factors, some of which were believed to be potential ARDS biomarkers [14,31,58]. Pneumonia and sepsis were found to be the top two etiological characters while their solid association with inflammation are inappropriate to be disregarded [59,60]. The current dilemma for ARDS diagnosis is that the low specificity and whether inflammatory factors can distinguish ARDS outcomes are both still in doubt [15]. Therefore, an ideal ARDS biomarker should precisely reflect pathophysiology features, e.g., alveolar injury or bronchial damage. The selection of pulmonary-originated epithelial biomarkers aimed to directly reflect lung injury and to reduce the heterogeneity brought by comorbidities. As shown in the subgroup analysis, SP-D distinguished ARDS patients from the pneumonia and sepsis patients. The Cohen’s d became more significant for the COVID-19 etiology group, and heterogeneity became lower for both the sepsis and COVID-19 etiology group. Nevertheless, due to the limited number of studies, it is regrettable that other biomarkers cannot be analyzed by the etiological subgroup. However, it is unignorable that some comorbidity may influence the diagnostic and prognostic value of biomarkers. Renal dysfunction may weaken the value of CC16 in the first 7 days but be regained in 28 days’ mortality [25]. Some cancer patients, such as myeloma and breast cancer, exhibited elevated serum KL-6 but with no evidence of pulmonary changes [61]. Therefore, biomarkers could be a powerful supplement to current criteria but not be an independent outcome predictor.

Previous meta-analysis recommended lung fluid biomarkers which is invasive and adds to patients’ suffering [31]. In this study, all included biomarkers were measured using the ELISA method based on blood, a non-invasive and more convenient test. A blood sample is the most universally applied in the clinical laboratory and serum is considered the gold standard in many diseases [60]. A commercialized ELISA kit presents a powerful and efficient technique with the advantage of stability, high sensitivity, and simple operation [62,63]. Meanwhile, quality control systems should be accurately built and availability, stability, and cross-reactivity of reagents should be tested when applying the ELISA method in the clinical laboratory [63]. Technically, the reported biomarkers are easily accessible with practical clinical laboratory applications. Biomarkers in parallel with the Berlin/AECC standard may solve the current obstacle of low specificity. 

This study updated the ARDS circulating pulmonary-originated epithelial biomarkers meta-analysis with a total of 2654 ARDS patients. Comparisons between normal subjects and patients with ARDS were rigorously excluded to reduce the heterogeneity brought by the control group. Compared to the previous meta-analysis of ARDS biomarkers, this study focuses on pulmonary proteins associated with pathophysiological features to better identify at-risk patients, which was proved in the subgroup analysis. To our knowledge, this is the first diagnostic test on ARDS biomarkers to calculate post-test probability and provide more supporting details for future clinical applications. Our study suggests that SP-D, KL-6, and CC16 are believed be potential biomarkers aiding the PaO_2_/FiO_2_ ratio in ARDS’ clinical identification and mortality prediction. However, the current study is limited to carrying out diagnostic accuracy tests for each biomarker. Future mechanism research should focus on the exact function of these proteins in different periods of ARDS and better explain the combined use of a set of biomarkers.

This meta-analysis has limitations. First, the amount of study for each biomarker was limited and may have an influence on the heterogeneity of the study. Measurable heterogeneity was exhibited in this study, and the random-effect model was applied in studies with more than 50% heterogeneity; nevertheless, our conclusion should be interpreted with caution. It has been proved in the subgroup study of SP-D that heterogeneity may come from etiology; however, we cannot confirm this conclusion on other biomarkers due to the lack of relevant research. Additionally, the APACHE score varied in different studies and patients which indicates the severity of disease. Second, the description of at-risk and reference standards was still different in included studies after restricted control groups, which is likely to bring a notable risk of bias. Third, the advancement in mechanical ventilation and extracorporeal membrane oxygenation (ECMO) over the 18 years of subject enrolment may have impacted on the mortality of ARDS patients. Furthermore, biomarker assays may have also changed during the 30 years enrolment span. ELISA kits from different corporations may also have brought different levels of detection which make it hard to accurately discuss the cut-off value. Fourth, the baseline sample moment was different in each study. ARDS is divided into an acute exudative phase combining diffuse alveolar damage and lung edema followed by a later fibroproliferative phase. The recorded sample moment ranges from immediately to 48 h after diagnosis, which may result in significant changes in the level of biomarkers. 

## 5. Conclusions

This study included 32 studies and 2654 ARDS patients and provided an overview of circulating pulmonary-originated epithelial protein biomarkers. After comprehensive assessment, SP-D, KL-6, and CC16 are promising biomarkers aiding the identification of at-risk patients and predictions of mortality which may help to clinically improve the ARDS diagnosis and outcome prediction. This study also reinforces the evidence of alveolar epithelial injury and bronchial cell injury as pathophysiological characteristics of ARDS. Future studies should focus on this field and provide more supporting details on the combined use of biomarkers and their precise function.

## Figures and Tables

**Table 1:** Demographic of patients included in the meta-analysis of ARDS at-risk patients’ identification.

Study	Biomarkers	Study Design	Setting	Study Population	ARDS Definition	Study Size	ARDS/ ALI (n)	Age	Male (%)	Plasma Sample Moment
Bersten 1998 [38]	SP-A/SP-B	Observational study	Single center	NM	NM	NM	10	NM	NM	NM
Briassoulis 2006 [22]	KL-6	Observational study	NM	Critically ill	AECC	36	9	8.4	NM	Within 8 H Of Admission
Buğra 2020 [40]	SP-D/KL-6	Observational study	Two centers	COVID-19 patient	Berlin	88	35	49	47	At admission
Determann 2009 [26]	SP-D/KL-6/CC16	Retrospective observational cohort	Single center	Critically ill	AECC	37	10	67	70	Baseline characteristics
Determann 2010 [41]	SP-D/KL-6/CC16	RCT	Multicenter	NM	AECC	36	16	58	50	At admission
Gao 2021 [13]	CC16	Case-control study	Single center	Critically ill	Berlin	200	100	56	58	NM
Gregory 1991 [19]	SP-A/SP-B	Observational study	Multicenter	NM	PaO_2_/ FiO_2_ ratio + clinic criteria	116	67	45	61	NM
Kropski 2009 [10]	CC16	Observational cohort	Single center	Critically ill	AECC	32	23	40	48	NM
Lin 2017 [47]	CC16	Retrospective observation cohort	Single center	Critically ill	Berlin	212	83	54	64	Within 2 h of admission
Liu 2014 [18]	SP-A	Observational study	Single center	Critically ill	AECC	60	9	1.9	NM	Within 24 h of diagnosis
Park 2017 [20]	SP-D	Retrospective observation cohort	Multicenter	Critically ill	Berlin	407	39	57	67	Within 48 h of ICU admission
Ren 2016 [39]	SP-A/CC16	prospective observational study	Single center	Critically ill	Berlin	212	83	54	67	Within 2 h of admission
Sato 2004 [23]	KL-6	Observational study	NM	Critically ill	AECC	47	28	40	36	At ICU admission or at the time of diagnosis
Todd 2010 [42]	SP-D	Prospective cohort study	NM	PICU	AECC + radiograph	1165	18	7	NM	A total of 12–18 h after ALI diagnosis
Tojo 2021 [43]	SP-D	Retrospective observational study	Single center	COVID-19 patient	Berlin	21	11	69	91	Day 1 in hospital
Villar 2021 [11]	SP-D	Observational study	Multicenter	Critically ill sepsis	Berlin	232	72	60	58	Within 24 h of diagnosis
Ware 2013 [44]	SP-D/CC16	Retrospective nested case-control study	Single center	Critically ill sepsis	AECC	200	100	56	52	On the morning of ICU day 2
Wu 2019 [48]	CC16	Clinical trial	Single center	Living-donor liver transplantation patient	Bilateral infiltrates + radiograph	73	13	59	46	At postoperative day 1
Yadav 2018 [45]	SP-D	Prospective observational case-control study	Single center	Adults undergoing elective thoracic, aortic vascular, or cardiac surgery	Berlin	467	26	63	77	Immediately follow the major intraoperative insult believed associated with development of lung injury
Ye 2019 [49]	CC16	Case-control study	Single center	Critically ill	Berlin	200	100	57	59	First day of ARDS diagnosis
Zeng 2019 [9]	SP-D/KL-6	Prospective cohort	Two centers	Critically ill	Berlin	99	49	53	73	At ICU admission
Zong 2017 [46]	SP-D	Prospective study	Single center	Critically ill sepsis	Berlin	88	48	NM	65	Within 24 h of ICU admission

**Table 2:** Demographic of patients included in the meta-analysis of ARDS mortality prediction.

Study	Mortality	Biomarkers	Study Design	Setting	Study Population	ARDS Definition	Study Size	ARDS/ALI (n)	Age	Male (%)	Plasma Sample Moment
Eisner 2003 [50]	38.5 (180D)	SP-A/D	RCT	Multicenter	NM	AECC + radiograph	565	565	51	59	Before implementing the ventilator protocol
Feng 2021 [53]	32.7 (28D)	CC16	Observational study	Single center	NM	Berlin	98	98	55	63	First day of ARDS diagnosis
Gao 2021 [13]	38 (hosp. mort.)	CC16	Case-control study	Single center	Critically ill	Berlin	200	100	56	58	NM
Han 2015 [51]	29.1 (28D)	SP-D	Observational study	Single center	Critically ill	AECC	79	79	NM	NM	Within 24 h of ARDS diagnosis
Kropski 2009 [10]	56.5 (ICU)	CC16	Observational cohort	Single center	Critically ill	AECC	32	23	40	48	At emergency department
Lesur 2006 [54]	37.2 (28D)	CC16	Prospective observational study	Multicenter	Critically ill	AECC	78	78	63	62	Within 48 h of ARDS diagnosis
Li 2021 [21]	30.8 (28D)	KL-6	Prospective cohort	Single center	Critically ill	Berlin	65	65	74	63	Day1 of RICU admission
Villar 2021 [11]	34.7 (28D)	SP-D/KL-6	Observational study	Multicenter	Critically ill sepsis	Berlin	232	72	60	58	Within 24 h of diagnosis
Ware 2013 [44]	27.3 (hosp. mort.)	SP-D	RCT	Multicenter	NM	AECC + radiograph	528	528	50	45	At time of enrolment (prior to randomization)
Yang 2017 [12]	44.9 (28D)	SP-D/KL-6	Observational study	Single center	Critically ill	Berlin	49	49	NM	NM	The day after ARDS diagnosis
Zeng 2019 [9]	34.7 (28D)	SP-D/KL-6	Prospective cohort	Two centers	Critically ill	Berlin	99	49	53	73	At time of ARDS diagnosis
Zhi 2016 [52]	30.7 (28D)	SP-A/SP-D/CC16	Retrospective study	Single center	Critically ill	AECC	101	101	56	46	Within 24 h of admission

**Table 3:** Demographic of ARDS patients included in the meta-analysis of CC16 diagnostic test accuracy.

Study	AUC	Study Design	Setting	Study Population	ARDS Definition	Study Size	ARDS/ ALI (n)	Age	Male (%)	Plasma Sample Moment
Determann 2009 [26]	0.91	Retrospective observational cohort	Single center	Critically ill	AECC	37	10	67	70	Baseline characteristics
Gao 2021 [13]	0.75	Case-control study	Single center	Critically ill	Berlin	200	100	56	58	NM
Lin 2020 [25]	0.87	Observational cohort	Single center	Critically ill	Berlin	479	83	52	58	Within 3 h of admission
Wang 2017 [55]	0.86	Prospective cohort	Multicenter	Critically ill sepsis	Berlin	100	59	NM	67	At admission
Ware 2013 [44]	0.60	Retrospective nested case-control study	Single center	Critically ill sepsis	AECC	200	100	56	52	On the morning of ICU day 2
Wu 2019 [48]	0.80	Clinical trial	Single center	Living-donor liver transplantation patient	Berlin	73	13	59	46	At postoperative day 1
Ye 2019 [49]	0.74	Case-control study	Single center	Critically ill	Berlin	200	100	57	59	First day of ARDS diagnosis

## Data Availability

Not applicable.

## Supplementary Materials

The following supporting information can be downloaded at: https://www.mdpi.com/article/10.3390/ijms24076090/s1.
