# Supplementary material for: Circulating Pulmonary-Originated Epithelial Biomarkers for Acute Respiratory Distress Syndrome: A Systematic Review and Meta-Analysis
Source: Int J Mol Sci. 2023 Mar 23;24(7):6090. doi: 10.3390/ijms24076090 (PMC10093822; doi:10.3390/ijms24076090)
Supplement: Supplementary file 1 [file ijms-24-06090-s001.zip › ijms-2238615-SI.pdf]

Additional file 1

Table of contents

Table S1. Extracted data(untransformed) from original article for ARDS identification.
Table S2. Extracted data(untransformed) from original article for mortality prediction.
Table S3. Extracted data(untransformed) from original article for mortality prediction.
Table S4. Etiology of ARDS patients.
Table S5. Sensitivity analysis of diagnosis prediction.
Table S6. Sensitivity analysis of mortality prediction.
Table S7. P value of Egger's test for biomarkers in continuous variables meta-analysis.

Figure of contents:

Figure. S1 Result of quality assessment.
Figure. S2 Forest plot of SMD for the association between diagnostic criteria subgroup and ARDS identification.
Figure. S3 Forest plot of SMD for the association between diagnostic criteria subgroup and ARDS mortality.
Figure. S4 Sensitivity analysis for CC16 diagnostic test.
Figure. S5 Funnel plots for biomarkers associated with ARDS diagnosis prediction.
Figure. S6 Funnel plots for biomarkers associated with ARDS mortality prediction.
Figure. S7 Deeks' funnel plot for CC16 diagnostic accuracy test.

Table S1 Extracted data(untransformed) from original article for ARDS identification

Biomarker	Study	Plasma concentration
SP-A		
	Berstern 1998	759±70 μg/ml
	Gregory 1991	29.88±8.49 μg/ml
	Liu 2014	95.32±5.43 mg/L
	Ren 2016	167.7±7.15 ng/ml
SP-B		
	Gregory 1991	0.57±0.24 μg/ml
	Berstern 1998	7174±1631 μg/ml
SP-D		
	Buğra 2020	82.3±45.4 ng/ml
	Determann 2009	12 (3.7, 28) ng/ml
	Determann 2010	275 (80, 462) ng/ml
	Park 2017	20.8 (12.7, 38.4) ng/ml
	Todd 2010	175 (109, 229) ng/ml
	Tojo 2021	16,596 (6733, 21397) pg/ml
	Villar 2021	7.65 (4.05, 20.07) ng/ml
	Ware 2013	86 (46, 159) ng/ml
	Yadav 2018	2.9 (1.4, 4.1) pg/ml
	Zeng 2019	6.8 (5.8, 8.4) ng/ml
	Zong 2017	80.9±28.3 g/l
KL-6		
	Briassoulis 2006	956±400 U/ml
	Determann 2009	292 (183,495) U/ml
	Determann 2010	477 (287,636) U/ml
	Sato 2004	537 (383,1119) U/ml
	Zeng 2019	3.5 (2.9, 4.0) ng/ml
CC16		
	Determann 2009	30 (10, 36) ng/ml
	Determann 2010	14.3 (9.0,19.0) ng/ml
	Gao 2021	38.78±14.70 mg/l
	Kropski 2009	22 (9, 44) ng/ml
	Lin2017	54.44±19.62 ng/ml
	Ware 2013	9.5 (5.0,15.8) ng/ml
	Wu 2019	27.5 ± 9.6 ng/ml
	Ye 2019	54.90±28.80 ng/l

Table S2 Extracted data(untransformed) from original article for mortality prediction

Biomarker	Study	Plasma concentration
SP-A		
	Eisner 2003	29 (14, 60) ng/ml
	Zhi 2016	24 (21, 29) pg/ml
SP-D		
	Eisner 2003	73 (33, 143) ng/ml
	Han 2015	16±7 ng/ml
	Villar 2021	8.39 (4.50, 17.71) ng/ml
	Ware 2010	92 (49, 192) ng/ml
	Yang 2017	2.6 (1.2, 6.3) mg/l
	Zeng 2019	8.5 (6.3, 13.2) ng/ml
	Zhi 2016	447 (267, 663) ng/ml
KL-6		
	Li 2021	801.4±261.1 U/ml
	Yang 2017	5667 (3326,8780) U/L
	Zeng 2019	5.0 (4.0,7.3) ng/ml
CC16		
	Feng 2021	44. 28±10. 73 ng/ml
	Gao 2021	30.52±9.47 ng/ml
	Kropski 2009	20 (10, 38) ng/ml
	Lesur 2006	8.9 (5.66, 26.38) µg/l
	Zhi 2016	162 (128, 208) ng/ml

Table S3 Extracted data from original article for CC16 diagnostic test accuracy

Study	Cut-off value	AUC	Sensitivity	Specificity
Wang 2017	27931.5074(NM)	0.861	0.746	0.878
Wu 2019	16.8 ng/ml	0.803	0.91	0.6
Ware 2013	NM	0.60	0.7	0.68
Determann 2009	18 ng/ml	0.91	0.8	0.92
Lin2020	32.77 ng/ml	0.868	0.817	0.8174
Gao 2021	32.95 mg/l	0.747	0.737	0.71
Ye 2019	33.67 ng/l	0.743	0.696	0.702

Table S4 Etiology of ARDS patients included in current study.

Etiology ARDS	Number(n)
Trauma	149
Sepsis	485
Pneumonia	832
Medication-related	50
Inhalation injury	4
Drowning	9
Cardiac arrest	2
Pancreatitis	35
Aspiration	204
Covid-19	43
End-stage liver disease	13
Other	348

Table S5 Sensitivity analysis of diagnosis prediction.

Biomarker	Study omitted	Estimate	[95% Conf. Interval]	
SP-A	Berstern 1998	3.51	-0.19	7.21
	Gregory 1991(1)	3.60	-0.22	7.42
	Liu 2014(2)	0.47	-1.63	2.56
	Ren 2016(3)	0.69	-1.46	-2.84
	Combined	1.78	-0.57	4.13
SP-D	Buğra 2020(4)	0.70	0.52	0.87
	Determann 2009(5)	0.73	0.60	0.87
	Determann 2010(6)	0.72	0.55	0.89
	Park 2017(7)	0.70	0.53	0.87
	Todd 2010(8)	0.70	0.54	0.86
	Tojo 2021(9)	0.70	0.54	0.85
	Villar 2021(10)	0.73	0.55	0.92
	Ware 2013(11)	0.71	0.52	0.90
	Yadav 2018(12)	0.75	0.61	0.89
	Zeng 2019(13)	0.70	0.52	0.87
	Zong 2017(14)	0.69	0.52	0.85
	Combined	0.71	0.56	0.87
KL-6	Briassoulis 2006(15)	0.98	0.37	1.59
	Determann 2009(5)	1.40	0.91	1.88
	Determann 2010(6)	1.19	0.37	2.02
	Sato 2004(16)	1.24	0.46	2.02
	Zeng 2019(13)	1.05	0.26	1.84
	Combined	1.17	0.55	1.79
CC16	Determann 2009(5)	0.77	-0.01	1.56
	Determann 2010(6)	0.74	-0.06	1.54
	Gao 2021(1)	0.60	-0.21	1.42
	Kropski 2009(17)	0.94	0.19	1.69
	Lin 2017(18)	0.56	-0.18	1.30
	Ware 2013(11)	0.96	0.43	1.49
	Wu 2019(19)	0.67	-0.13	1.47
	Ye 2019(20)	0.68	-0.21	1.58
	Combined	0.74	0.01	1.46

Table S6 Sensitivity analysis of prognosis prediction.

Biomarker	Study omitted	Estimate	[95%Conf.Interval]	
SP-D	Eisner 2003(21)	0.68	0.19	1.17
	Han 2015(22)	0.70	0.31	1.08
	Villar 2021(10)	0.71	0.34	1.09
	Ware 2010(11)	0.67	0.18	1.16
	Yang 2017(23)	0.57	0.20	0.95
	Zeng 2019(13)	0.59	0.22	0.97
	Zhi 2016(24)	0.40	0.22	0.59
	Combined	0.62	0.27	0.96
KL-6	Li 2021(25)	0.82	0.39	1.24
	Yang 2017(23)	0.94	0.53	1.36
	Zeng 2019(13)	0.82	0.42	1.22
	Combined	0.86	0.53	1.20
CC16	Feng 2021(26)	0.73	0.35	1.10
	Gao 2021(1)	0.85	0.18	1.52
	Kropski 2009(17)	1.07	0.54	1.59
	Lesur 2006(27)	1.03	0.45	1.61
	Zhi 2016(24)	0.94	0.27	1.61
	Combined	0.92	0.42	1.43

Table S7 *P* value of Egger's test for biomarkers in continuous variables meta-analysis.

Biomarker	*P* value
Diagnosis prediction	
SP-A	0.25
SP-D	0.94
KL-6	0.78
CC16	0.83
Mortality prediction	
SP-D	0.37
KL-6	0.96
CC16	0.56

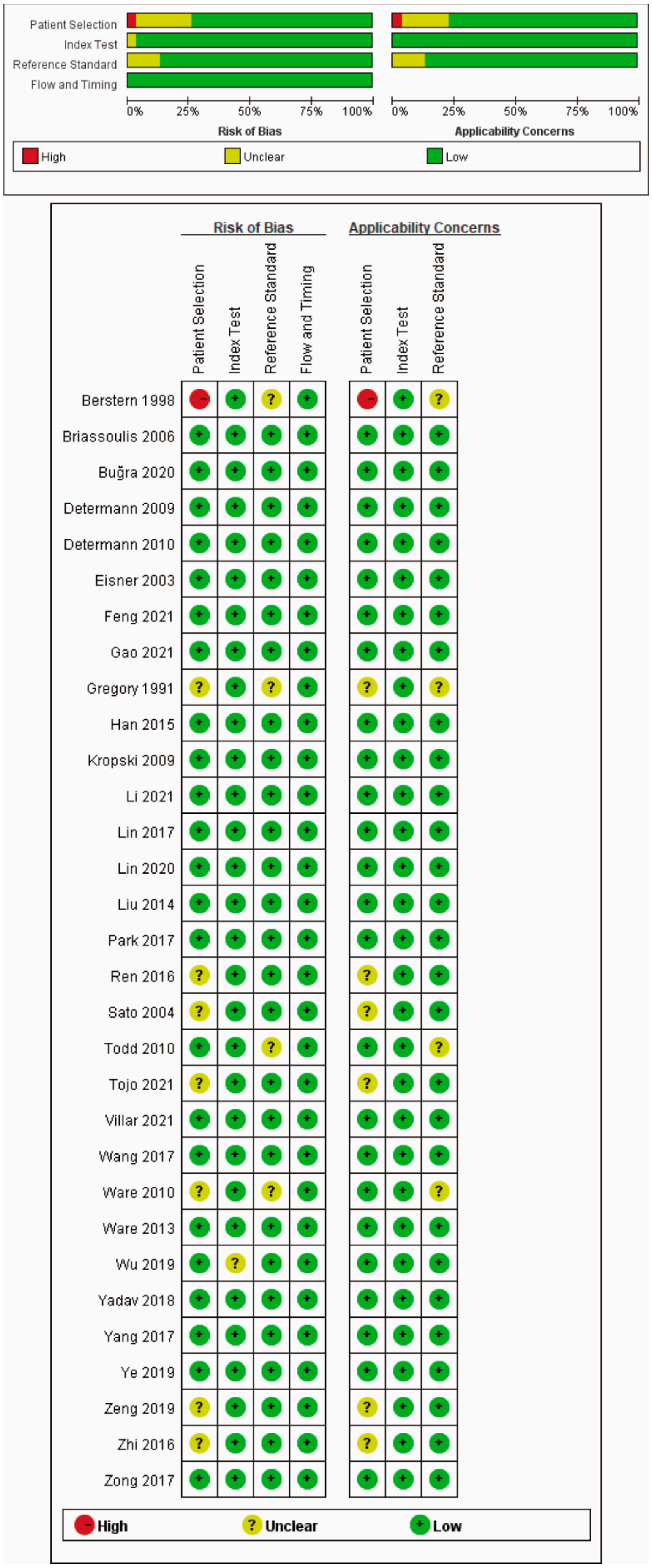

Figure. S1 Quality assessment of included studies.

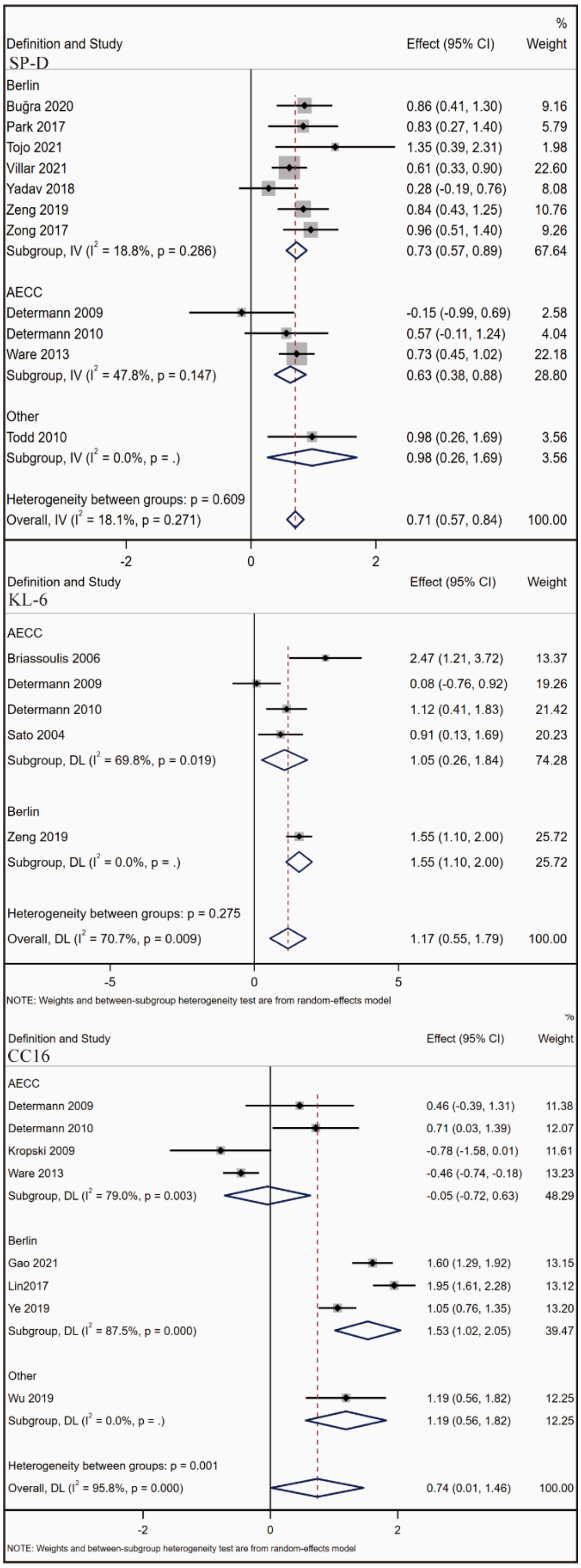

Figure. S2 Forest plot of SMD for the association between diagnostic criteria subgroup and ARDS identification.

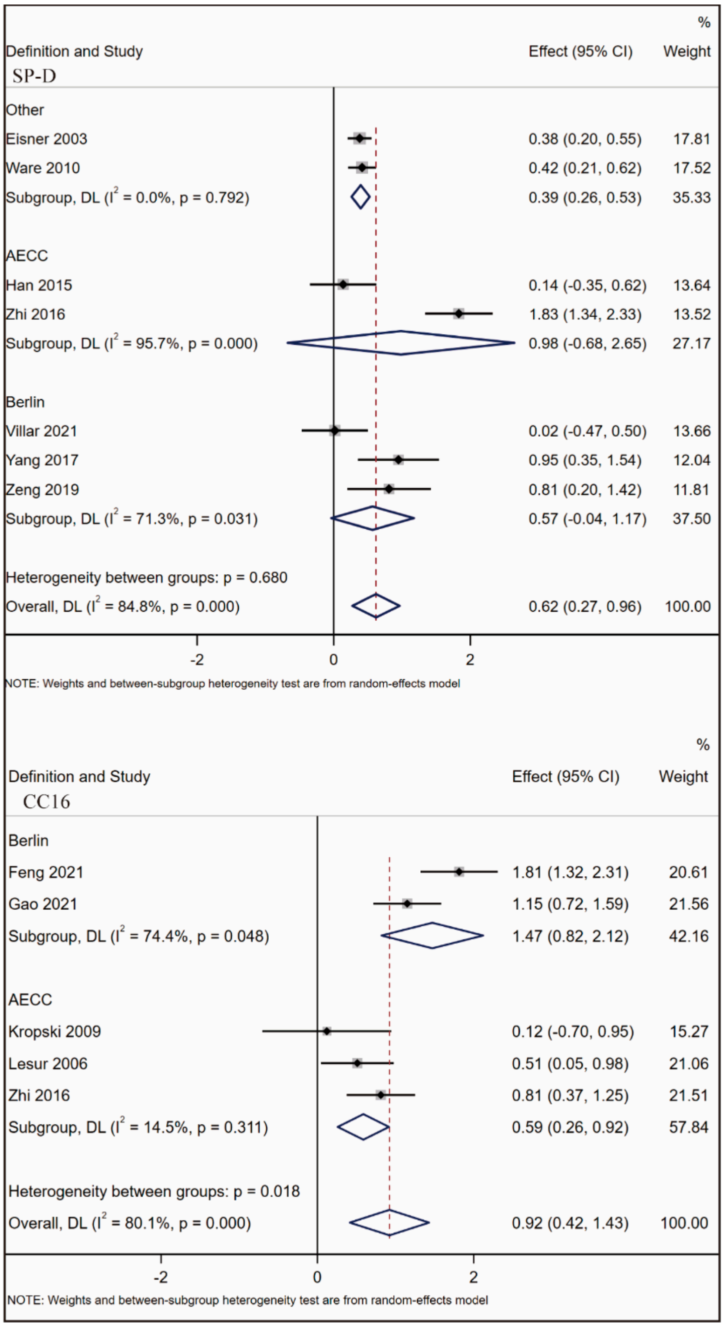

Figure. S3 Forest plot of SMD for the association between diagnostic criteria subgroup and ARDS mortality.

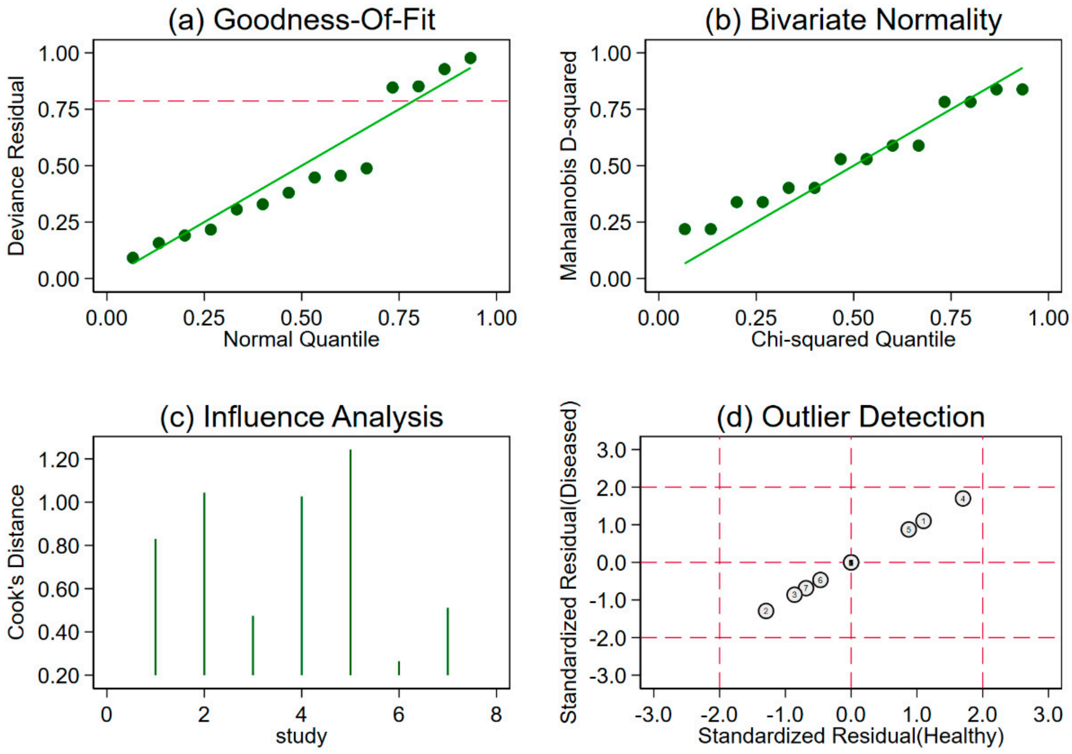

Figure S4. Sensitivity analysis for CC16 diagnostic test.

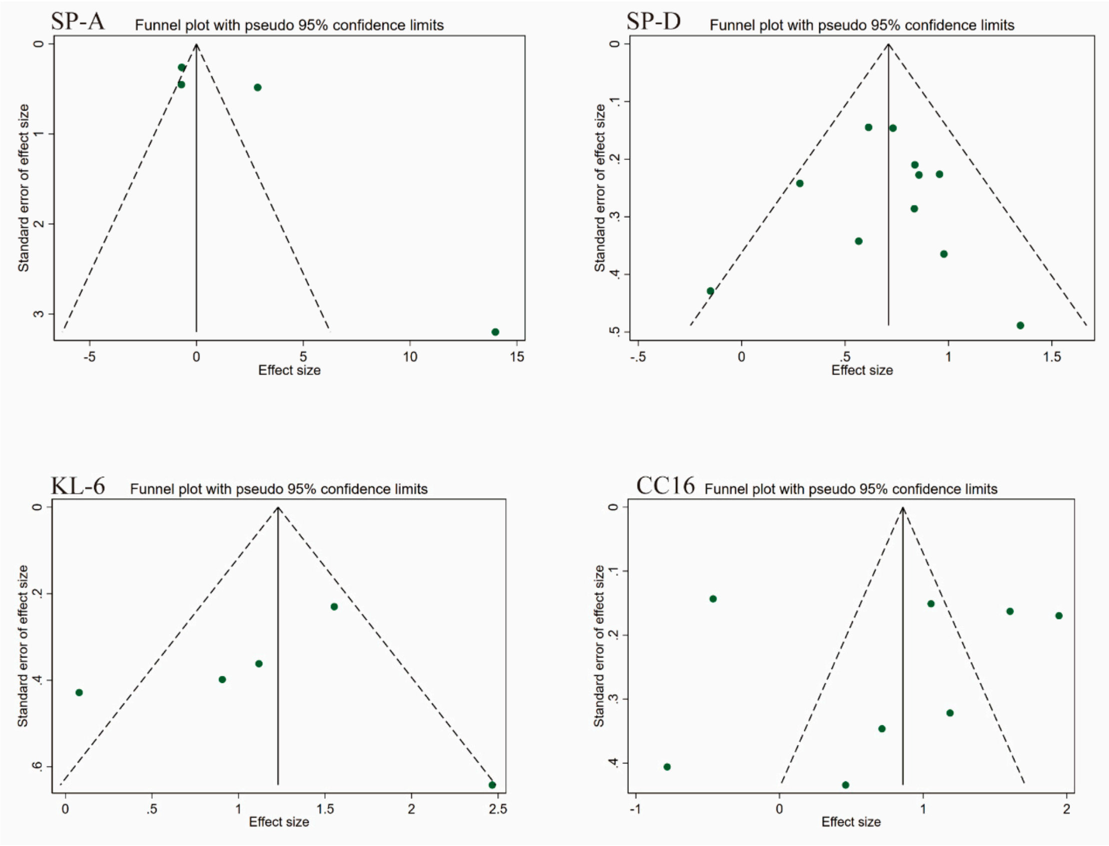

Figure S5. Funnel plots for biomarkers associated with ARDS diagnosis prediction.

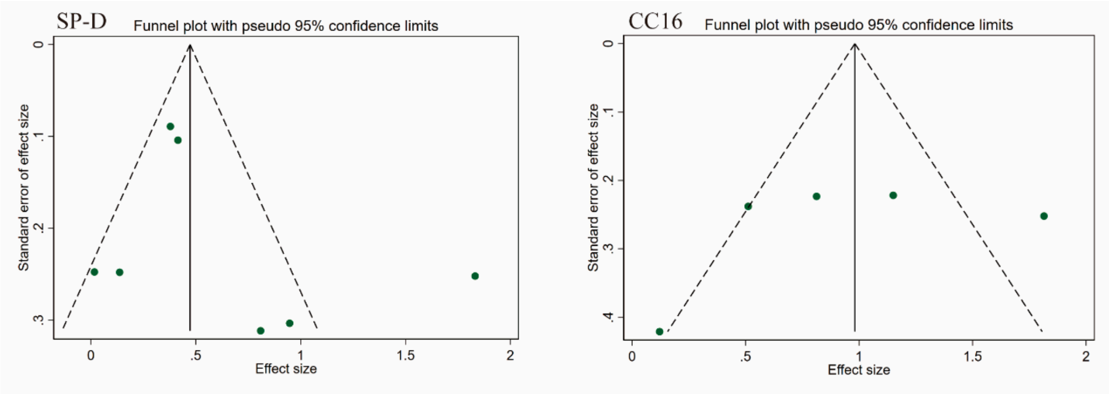

Figure S6. Funnel plots for biomarkers associated with ARDS mortality prediction.

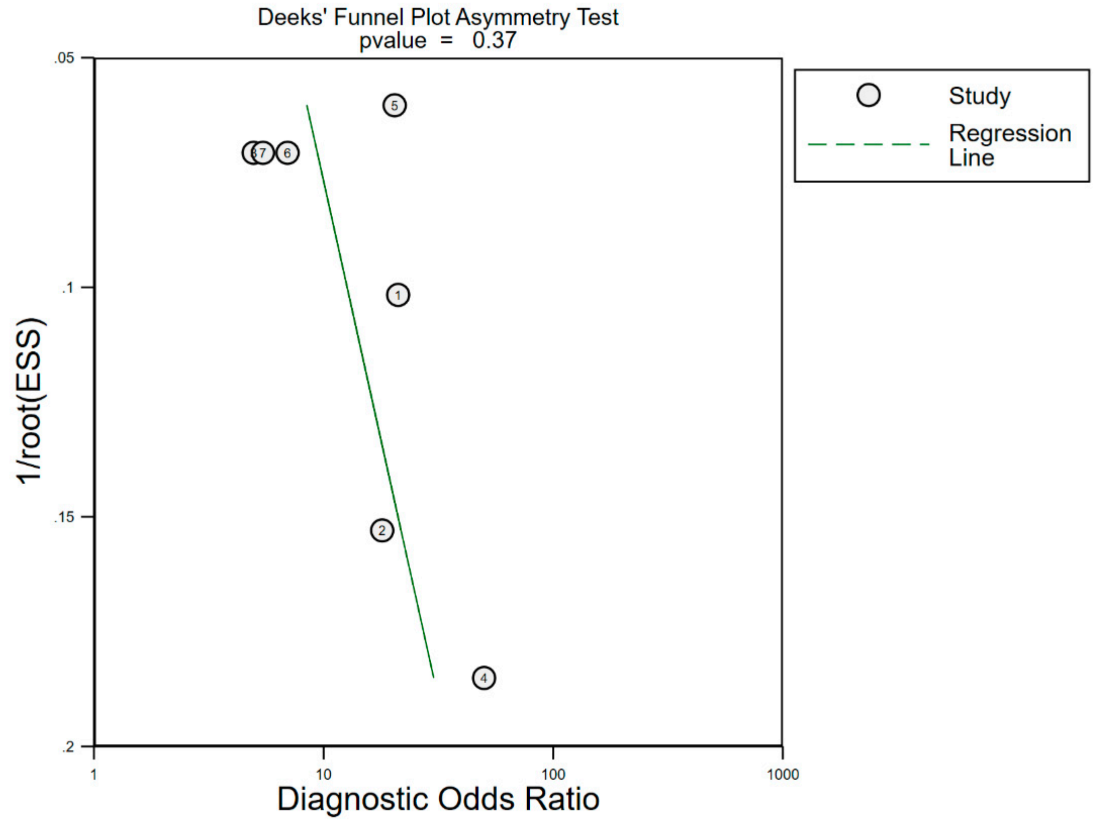

Figure S7. Deeks' funnel plot for CC16 diagnostic accuracy test.

1. Gao Y, Li J. [Significance of combined application of biomarkers in the diagnosis and prognosis assessment of patients with acute respiratory distress syndrome]. Zhonghua wei zhong bing ji jiu yi xue. 2021;33(1):69-73.
2. Liu X, Shao Q, Bai X, Sun R, Liu P. The early diagnosis value of the serum surfactant protein a in critically ill acute lung injury of HFMD. Chongqing Medicine. 2014;43(6):663-5.
3. Ren S, Chen X, Jiang L, Zhu B, Jiang Q, Xi X. Deleted in malignant brain tumors 1 protein is a potential biomarker of acute respiratory distress syndrome induced by pneumonia. Biochemical and biophysical research communications. 2016;478(3):1344-9.
4. Kerget B, Kerget F, Koçak AO, Kızıltunç A, Araz Ö, Uçar EY, et al. Are Serum Interleukin 6 and Surfactant Protein D Levels Associated with the Clinical Course of COVID-19? Lung. 2020;198(5):777-84.
5. Determann RM, Millo JL, Waddy S, Lutter R, Garrard CS, Schultz MJ. Plasma CC16 levels are associated with development of ALI/ARDS in patients with ventilator-associated pneumonia: a retrospective observational study. BMC pulmonary medicine. 2009;9:49.
6. Determann RM, Royakkers AA, Haitsma JJ, Zhang H, Slutsky AS, Ranieri VM, et al. Plasma levels of surfactant protein D and KL-6 for evaluation of lung injury in critically ill mechanically ventilated patients. BMC pulmonary medicine. 2010;10:6.
7. Park J, Pabon M, Choi AMK, Siempos, II, Fredenburgh LE, Baron RM, et al. Plasma surfactant protein-D as a diagnostic biomarker for acute respiratory distress syndrome: validation in US and Korean cohorts. BMC pulmonary medicine. 2017;17(1):204.
8. Todd DA, Marsh MJ, George A, Henderson NG, Barr H, Sebastian S, et al. Surfactant phospholipids, surfactant proteins, and inflammatory markers during acute lung injury in children. Pediatric critical care medicine : a journal of the Society of Critical Care Medicine and the World Federation of Pediatric Intensive and Critical Care Societies. 2010;11(1):82-91.
9. Tojo K, Yamamoto N, Mihara T, Abe M, Goto T. Distinct temporal characteristics of circulating alveolar epithelial and endothelial injury markers in ARDS with COVID-19. Critical care (London, England). 2021;25(1):169.
10. Villar J, Herrán-Monge R, González-Higueras E, Prieto-González M, Ambrós A, Rodríguez-Pérez A, et al. Clinical and biological markers for predicting ARDS and outcome in septic patients. Scientific reports. 2021;11(1):22702.
11. Ware LB, Koyama T, Zhao Z, Janz DR, Wickersham N, Bernard GR, et al. Biomarkers of lung epithelial injury and inflammation distinguish severe sepsis patients with acute respiratory distress syndrome. Critical care (London, England). 2013;17(5):R253.
12. Yadav H, Bartley A, Keating S, Meade LA, Norris PJ, Carter RE, et al. Evolution of Validated Biomarkers and Intraoperative Parameters in the Development of Postoperative ARDS. Respiratory care. 2018;63(11):1331-40.
13. Zeng X, Lu D, Zhang X, Li J. Predictive and prognostic evaluation of five serum markers for acute respiratory distress syndrome. Journal of Xi'an Jiaotong University Medical Sciences. 2019;40(4):588-92,618.
14. Zong X, Li Z, Wei D, Chen Y, Sun G. Predictive and prognostic evaluation of surfactant protein D,von Willebrand factor and interleukin-8 for sepsis-induced acute respiratory distress syndrome. Chinese Journal of Clinical Laboratory Science. 2017;35(2):118-21.
15. Briassoulis G, Mavrikiou M, Margeli A, Lazaropoulou C, Natsi L, Papassotiriou I, et al. Circulating

levels of KL-6 in acute respiratory distress syndrome sepsis or traumatic brain injury in critically ill children. Pediatric pulmonology. 2006;41(8):790-5.
16. Sato H, Callister ME, Mumby S, Quinlan GJ, Welsh KI, duBois RM, et al. KL-6 levels are elevated in plasma from patients with acute respiratory distress syndrome. The European respiratory journal. 2004;23(1):142-5.
17. Kropski JA, Fremont RD, Calfee CS, Ware LB. Clara cell protein (CC16), a marker of lung epithelial injury, is decreased in plasma and pulmonary edema fluid from patients with acute lung injury. Chest. 2009;135(6):1440-7.
18. Lin J, Zhang W, Wang L, Tian F. Diagnostic and prognostic values of Club cell protein 16 (CC16) in critical care patients with acute respiratory distress syndrome. Journal of clinical laboratory analysis. 2018;32(2).
19. Wu CY, Cheng YJ, Hung MH, Lin IJ, Sun WZ, Chan KC. Association between Early Acute Respiratory Distress Syndrome after Living-Donor Liver Transplantation and Perioperative Serum Biomarkers: The Role of Club Cell Protein 16. BioMed research international. 2019;2019:8958069.
20. Ye J, Lin J, Zhong C, Ye J, Shi M, Wei J, et al. The clinical value of combined detection of serum angiopoietin 2 and Clara cell protein 16 in the early diagnosis of acute respiratory distress syndrome. Chinese Journal of Emergency Medicine. 2019;28(9):1112-7.
21. Eisner MD, Parsons P, Matthay MA, Ware L, Greene K. Plasma surfactant protein levels and clinical outcomes in patients with acute lung injury. Thorax. 2003;58(11):983-8.
22. Han F, Wang H, Ni Y. Dynamic Changes of Lung Surfactant Protein D in Patients With Acute Respiratory Distress Syndrome. Chinese General Practice. 2015;18(13):1541-4.
23. Yang Q, Li Z, Lan H, Xiong S, Wang S, Yan C. Research of the biomarkers in pulmonary and extrapulmonary acute respiratory distress syndrome. National Medical Journal of China. 2017;97(26):2023-7.
24. Zhi D, Duan M, Liu Z, Li A. Surfactant, pulmonary-associated protein D may serve as a biomarker in addition to APACHE II score in predicting mortality rate of ARDS. International Journal of Clinical and Experimental Pathology. 2016;9(2):2127-33.
25. Li S, Gao Y, Zhou L, Li X, Zhang L, Dong R, et al. The prognosis evaluation of sICAM-1,KL-6 combined with EVLWI in severe pneumonia patients with acute respiratory distress syndrome. Chinese Journal of Emergency Medicine. 2021;30(6):730-6.
26. Feng X, Huang Y, Chen Y, Fu Z, Xu J, Hao J. Prognostic value of serum Activin-A CC-16 and IL-18 levels in the patients with acute respiratory distress syndrome. Chinese Journal of Critical Care Medicine. 2021;41(1):41-5.
27. Lesur O, Langevin S, Berthiaume Y, Légaré M, Skrobik Y, Bellemare JF, et al. Outcome value of Clara cell protein in serum of patients with acute respiratory distress syndrome. Intensive Care Med. 2006;32(8):1167-74.

Additional file 2

Part of the content was tailored to better fit the study. Signalling question in patient

selection, “Was a case-control design avoided?” was replaced by “Were specific include and exclude criteria mentioned?” because case-control studies were inappropriate to be neglected in our study design. In index tests, the question of “If a threshold was used, was it pre-specified?” would not be answered unless studies were included for diagnostic test accuracy.

The exact QUADAS-2 tool used in this meta-analysis was listed below.

1. Patient selection

A. Risk of Bias

Was a consecutive or random sample of patients enrolled?

Yes / No / Unclear

Did the study avoid inappropriate exclusions?

Yes / No / Unclear

Were there clear inclusion and exclusion criteria?

Yes / No / Unclear

Could the selection of patients have introduced bias?

High risk / Unclear risk / Low risk

B. Concerns regarding applicability

Are there concerns that the included patients and setting do not match the review question?

High concern / Unclear concern / Low concern

2. All tests

A. Risk of Bias

Were the index test results interpreted without knowledge of the results of the reference standard?

Yes / No / Unclear

If a threshold was used, was it pre-specified?

Yes / No / Unclear

Could the conduct or interpretation of the index test have introduced bias?

High risk / Unclear risk / Low risk

B. Concerns regarding applicability

Are there concerns that the index test, its conduct, or interpretation differ from the review question?

High concern / Unclear concern / Low concern

3. Reference Standard

A. Risk of Bias

Is the reference standards likely to correctly classify the target condition?

Yes / No / Unclear

Were the reference standard results interpreted without knowledge of the results of the index tests?

Yes / No / Unclear

Could the reference standard, its conduct, or its interpretation have introduced bias?

High risk / Unclear risk / Low risk

B. Concerns regarding applicability

Are there concerns that the target condition as defined by the reference standard does not match the question?

High concern / Unclear concern / Low concern

4. Flow and Timing

A. Risk of Bias

Was there an appropriate interval between index test and reference standard?

Yes / No / Unclear

Did all patients receive the same reference standard?

Yes / No / Unclear

Were all patients included in the analysis?

Yes / No / Unclear

Could the patient flow have introduced bias?

High risk / Unclear risk / Low risk

Annotations: "Reference standard" in this meta-analysis implies criteria used for acute respiratory distress syndrome diagnosis, including American European Consensus Conference criteria, lung injury score and so on. **"Flow and timing"** is a part of evaluating possible bias of the process, including patients recruiting and biomarker measurement.

Additional file 3

Search strategy: date-31th December 2021

Pubmed: ((acute lung injury) OR (ALI) OR (ARDS) OR (acute respiratory distress syndrome)) AND ((club cell secretory protein 10) OR (clara cell secretory protein 10) OR (CC10) OR (club cell secretory protein 16) OR (clara cell secretory protein 16) OR (CC16) OR (krebs von den lungen-6) OR (KL-6) OR (surfactant protein A) OR (SP-A) OR (surfactant protein B) OR (SP-B) OR (surfactant protein C) OR (SP-C) OR (surfactant protein D) OR (SP-D))
1119

Web of science: TS=(acute lung injury OR ALI OR ARDS OR acute respiratory distress syndrome) AND TS=(club cell secretory protein 10 OR clara cell secretory protein 10 OR CC10 OR club cell secretory protein 16 OR clara cell secretory protein 16 OR CC16 OR krebs von den lungen-6 OR KL-6 OR surfactant protein A OR SP-A OR surfactant protein B OR SP-B OR surfactant protein C OR SP-C OR surfactant protein D OR SP-D)
2013

Ovid: (acute lung injury$ OR ALI$ OR ARDS$ OR acute respiratory distress syndrome) AND (club cell secretory protein 10$ OR clara cell secretory protein 10$ OR CC10$ OR club cell secretory protein 16$ OR clara cell secretory protein 16$ OR CC16$ OR $krebs von den lungen-6$ OR KL-6$ OR surfactant protein A$ OR SP-A$ OR surfactant protein B$ OR SP-B$ OR surfactant protein C$ OR SP-C$ OR surfactant protein D$ OR SP-D)
2492

Embase: ('acute lung injury'/exp OR 'acute lung injury' OR (acute AND ('lung'/exp OR lung) AND ('injury'/exp OR injury)) OR ali OR 'ards'/exp OR ards OR 'acute respiratory distress syndrome'/exp OR 'acute respiratory distress syndrome' OR (acute AND ('respiratory'/exp OR respiratory) AND ('distress'/exp OR distress) AND ('syndrome'/exp OR syndrome))) AND ('club cell secretory protein 10' OR (club AND ('cell'/exp OR cell) AND secretory AND ('protein'/exp OR protein) AND 10) OR 'clara cell secretory protein 10' OR (clara AND ('cell'/exp OR cell) AND secretory AND ('protein'/exp OR protein) AND 10) OR cc10 OR 'club cell secretory protein 16'/exp OR 'club cell secretory protein 16' OR (club AND ('cell'/exp OR cell) AND secretory AND ('protein'/exp OR protein) AND 16) OR 'clara cell secretory protein 16'/exp OR 'clara cell secretory protein 16' OR (clara AND ('cell'/exp OR cell) AND secretory AND ('protein'/exp OR protein) AND 16) OR cc16 OR 'krebs von den lungen-6' OR (krebs AND von AND den AND 'lungen 6') OR 'kl 6'/exp OR 'kl 6' OR 'surfactant protein a'/exp OR 'surfactant protein a' OR (('surfactant'/exp OR surfactant) AND ('protein'/exp OR protein) AND a) OR 'sp a' OR 'surfactant protein b'/exp OR 'surfactant protein b' OR (('surfactant'/exp OR surfactant) AND ('protein'/exp OR protein) AND b) OR 'sp b' OR 'surfactant protein c'/exp OR 'surfactant protein c' OR (('surfactant'/exp OR surfactant) AND ('protein'/exp OR protein) AND c) OR 'sp c' OR 'surfactant protein d'/exp OR 'surfactant protein d' OR (('surfactant'/exp OR surfactant) AND ('protein'/exp OR protein) AND d) OR 'sp d')
2869

Cochrane Library

ID	Search	Hits
#1	acute lung injury	2710
#2	ALI	6050
#3	ARDS	2377
#4	acute respiratory distress syndrome	3287
#5	club cell secretory protein 10	10
#6	clara cell secretory protein 10	23
#7	CC10	16
#8	club cell secretory protein 16	6
#9	clara cell secretory protein 16	15
#10	CC16	53
#11	krebs von den lungen-6	16
#12	KL-6	67
#13	surfactant protein A	281
#14	SP-A	1119
#15	surfactant protein B	108
#16	SP-B	43
#17	surfactant protein C	145
#18	SP-C	43
#19	surfactant protein D	190
#20	SP-D	120
#21	#1 or #2 or #3 or #4	10826
#22	#5 or #6 or #7 or #8 or #9 or #10 or #11 or #12 or #13 or #14 or #15 or #16 or #17 or #18 or #19	1493
#23	#21 and #22	119
